# Platelet factor 4 inhibits human hair follicle growth and promotes androgen receptor expression in human dermal papilla cells

**DOI:** 10.7717/peerj.9867

**Published:** 2020-09-04

**Authors:** Ke Sha, Mengting Chen, Fangfen Liu, San Xu, Ben Wang, Qinqin Peng, Yiya Zhang, Hongfu Xie, Ji Li, Zhili Deng

**Affiliations:** 1 Department of Dermatology, Xiangya Hospital, Central South University, Changsha, China; 2 National Clinical Research Center for Geriatric Disorders, Xiangya Hospital, Central South University, Changsha, China; 3 Key Laboratory of Molecular Radiation Oncology Hunan Province, Central South University, Changsha, China; 4 Key Laboratory of Organ Injury, Aging and Regenerative Medicine of Hunan Province, Central South University, Changsha, China; 5 Xiangya International Academy of Translational Medicine, Central South University, Changsha, China

**Keywords:** Hair loss, Hair follicle, Platelet-rich plasma, Platelet factor 4, Androgen receptor, Dermal papilla cells

## Abstract

Platelet-rich plasma (PRP) has been reported recently as a potential therapeutic approach for alopecia, such as androgenetic alopecia, but the exact mechanisms and effects of specific components of this recipe remain largely unknown. In this study, we identified that platelet factor 4 (PF4), a component of PRP, significantly suppressed human hair follicle growth and restrained the proliferation of human dermal papilla cells (hDPCs). Furthermore, our results showed that PF4 upregulated androgen receptor (AR) in human dermal papilla cells *in vitro* and via hair follicle organ culture. Among the hair growth-promoting and DP-signature genes investigated, PF4 decreased the expression of *Wnt5a*, *Wnt10b*, *LEF1, HEY1* and *IGF-1,* and increased* DKK1* expression*,* but did not affect *BMP2* and *BMP4* expression. Collectively, Our data demonstrate that PF4 suppresses human hair follicle growth possibly via upregulating androgen receptor signaling and modulating hair growth-associated genes, which provides thought-provoking insights into the application and optimization of PRP in treating hair loss.

## Introduction

The hair follicle (HF) is able to repeatedly go through cycles of degeneration (catagen), rest (telogen), and regeneration (anagen) over all adult life. The hair follicle cycle depends on the capability of HF stem cells (HFSCs), residing in the bulge, to temporarily escape from their quiescent state to start the anagen phase. The activities of HFSCs are mainly regulated by the dermal papilla (DP) in the bottom of hair follicles (Deng et al., 2015; Schneider, Schmidt-Ullrich & Paus, 2009). The DPCs function as the signaling center to govern the behaviors of HFSCs and their descendent cells to achieve hair regeneration (Morgan, 2014). Numerous signals from DP, such as Wnt/*β*-catenin and BMP signals have been shown to be essential for the hair growth (Deng et al., 2015; Plikus, 2012; Tang et al., 2016; Li et al., 2012).

Various hair loss disorders are mainly featured by the incapability of rebooting the growth phase of the hair follicle cycle. In androgenetic alopecia (AGA), elevated androgen receptor (AR) in DP cells is one of the causal factors for AGA (Premanand & Reena, 2018). The dysregulation of androgen receptor signaling affects the production of hair growth-associated paracrine signals in the DP cells. Disruption of these signals hampers HFSCs proliferation and differentiation, causing anagen shortening, thus leading to progressive miniaturization of hair follicles, which is a major feature of AGA (Ceruti, Leiros & Balana, 2018; Qi & Garza, 2014; Shin et al., 2013; Kwack et al., 2008). As a consequence, DP cells are considered as the major therapy target for AGA. The existing therapies are mostly designed to restrain further hair loss, and the efficacy of new hair growth with these therapies are not dissatisfactory and obviously ameliorating effect is not always achieved (Stevens & Khetarpal, 2019).

PRP is a derivative of whole blood, also known as autologous conditioned plasma (Mussano et al., 2016). PRP was originally applied to the medical field as a promising hemostatic approach suitable for surgical setting and wound healing (Cieslik-Bielecka et al., 2012; Nicoli et al., 2015). Recently, this technique has been explored in the field of dermatology including wound healing, scar revision, skin rejuvenation, fat graft and so on (Emer, 2019). Furthermore, PRP has also been demonstrated as an efficient cure to treat hair disorders, such as androgenetic alopecia, owing to its autologous, least injuries, less adverse effects, and more acceptable cost in comparison to hair implantation (Strazzulla et al., 2018). Evidence indicates that when injected into scalp, platelets in PRP become activated and secrete a series of cytokines andgrowth factors , like insulin like growth factor-1 (IGF-1), platelet-derived growth factor and epidermal growth factor from their alpha granules, which may promote hair growth (Mussano et al., 2016; Emer, 2019; Cervantes et al., 2018). However, the exact mechanisms and roles of specific components of PRP remain largely unresolved.

PF4, as an important member of the CXC chemokine family of small proteins, is one of the richest factors secreted to plasma after blood platelets activation. Even then, its level in plasma is only up to the nanogram level per milliliter (Gleissner, Von Hundelshausen & Ley, 2008; Chen et al., 2020; Makarewicz-Wujec et al., 2020). It has been reported to have an antiproliferative effect on fibroblasts and endothelial cells, but there is no report about its role in human hair growth. Here, we demonstrated that PF4, a component of PRP, notably suppressed human hair follicle growth and inhibited the proliferation of human derma papilla cells. Mechanically, we showed that PF4 promoted the expression of AR in human dermal papilla cells *in vitro* and via hair follicle organ culture. Among the hair growth-promoting and DP-signature genes investigated, PF4 reduced the expression of *Wnt5a*, *Wnt10b*, *LEF1, HEY1* and *IGF-1,* and increased the expression of *DKK1,* but did not alter the expression of *BMP2* and *BMP4*. Taken together, Our findings reveal that PF4 inhibits human hair follicle growth possibly by enhancing androgen receptor signaling and decreasing hair growth-promoting genes expression, providing new insights into the application and optimization of PRP in the administration of hair loss.

## Material and Methods

### Human hair follicle isolation and culture

Scalp biopsies were acquired from occipital site of AGA individuals experiencing hair grafting surgery. The study was permitted by the Institutional Review Board of Xiangya hospital (IRB NO. 201611609), and informed consent from participants was obtained. The human hair follicles were isolated and cultured as previously described (Luo et al., 2018). Specifically, hair follicles were incubated with or without PF4 (R&D system, USA), and were photographed every two days. 90 hair follicles in anagen phase were obtained from 3 volunteers and incubated with different concentrations of PF4, and the measurements were repeated for triplicate with 30 hair follicles in total for each dose group.

### hDPCs isolation and culture

The human DPCs were acquired and cultured according to previous strategy (Gledhill, Gardner & Jahoda, 2013). hDPCs at passages 2-5 were used in the present study. The hDPCs were digested with 0.1% trypsin and counted with a cell counter (JIMBIO FIL, China).

### RNA collection and qPCR

Data were collected as previously described (Luo et al., 2018). Specifically, total RNA was acquired by TRIzol RNA extraction reagent (Thermo Fisher Scientific, USA). cDNA was obtained using the PrimeScript™ RT reagent Kit (Takara, Japan) according to the manufacture’s instruction. qPCR was performed with SYBR Green Supermix (Bio-Rad, USA). The relative mRNA expression levels were figured by employing the delta CT means relative to GAPDH. The fold change for genes was normalized to control cell cultures. All primers for PCR are indicated in Table S1.

### Western blot assays

All procedures for western blot were conducted as previously described (Deng et al., 2019). Primary antibodies used were rabbit anti-Androgen receptor (1:1000, Cell Signaling Technology, USA) and mouse anti-Tubulin (1:1000, Cell Signaling Technology, USA).

### Immunofluorescence

Immunofluorescence of cultured HFs was performed as previously described (Chen et al., 2019). Specifically, hair follicle frozen sections were fixed for ten min by paraformaldehyde (PFA), washed with PBS, and blocked for 60 min in blocking buffer (5% normal donkey serum supplemented with 0.3% Triton X-100). Primary antibodies were incubated for 12–16 h at 4 °C. Followed by washed with PBS, sections were stained with Alexa Fluor 488/594-tagged secondary antibodies (Thermo Fisher Scientific, USA) for 1 h at room temperature, then counterstained with nuclear staining reagent DAPI. Pictures were taken with a Zeiss Axioplan 2 microscope. Primary antibodies used were rabbit anti-Androgen receptor (1:200, Cell SignalingTechnology, USA), rabbit anti-ki67 (1:500, Cell SignalingTechnology, USA) and mouse anti-K15 (1:500, Thermo Fisher Scientific, USA).

### ELISA

The hDPCs were incubated in 6-well plate (1 ml/well) after counted 50 cells/ µl. DP cells were subjected into three groups, after cultured 48 h with PF4. Corresponding kits were used to test the bioactive factors of the supernatant. Wnt5a, IGF1 and BMP2 were measured by Wnt5a, IGF1 and BMP2 ELISA kits (CUSABIO) following the manufacturer’s instruction, respectively.

### Statistical analysis

Statistical analysis was performed by using GraphPad 8.0 and SPSS 18.0. Data are displayed as the mean ± SEM. Statistical significance (*P < 0.05, **P < 0.01) was calculated using 2-tailed unpaired Student’s *t*-test for the comparisons between two groups.

## Results

### PF4 suppresses hair growth in cultured human HFs

**Figure 1:**
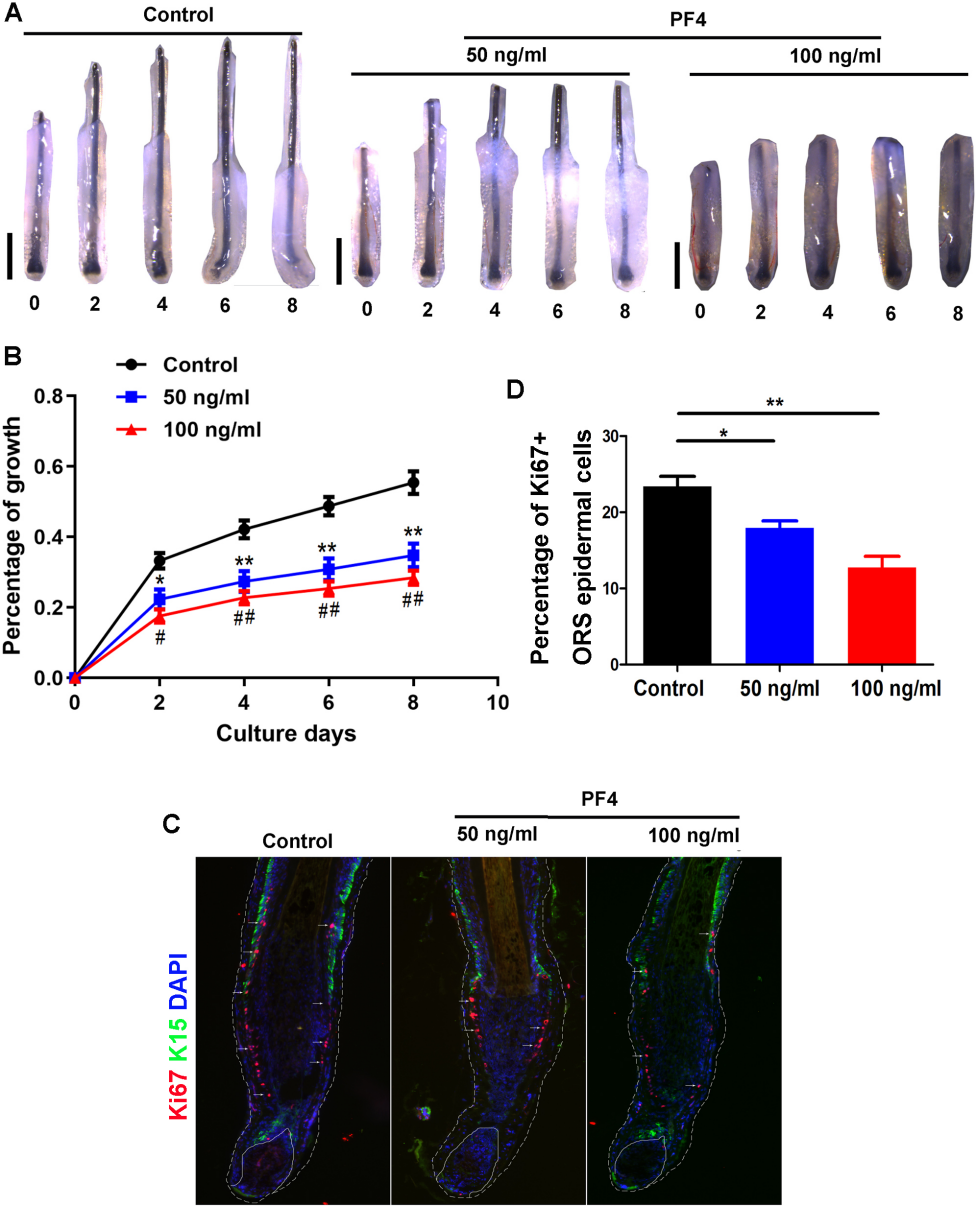
PF4 suppresses the elongation of hair shaft in cultured human hair follicles. Isolated human scalp hair follicles were cultured for 8 days in the presence of different doses of PF4. (A) Typical pictures of the hairs at day 0, 2, 4, 6, 8. (B) Data are presented as the percentage of growth (relative to day 0) of the hair follicles treated with PF4 (0, 50, 100 ng/ml). (C) immunostaining of Ki67 in hair follicles treated with PF4 (0, 50, 100 ng/ml) for 8 days. (D) Percentage of Ki67 positive cell in ORS of hair follicles treated with PF4. Data are reported as mean + SEM. Student’s *t*-test was used to compare data. * *P* < 0.05,** *P* < 0.01, comparation between 0 and 50 ng/ml. # *P* < 0.05, ## *P* < 0.01, comparation between 0 and 100 ng/ml. Scale bar = 1 mm.

To explore the effect of PF4, a component of PRP (Hattori & Ishihara, 2017), on hair growth, human HFs were acquired and incubated with or without PF4. The concentration of PF4 (0, 50, 100 ng/ml) was commonly used as previously described, and similar to or slightly higher than that in healthy human plasma, which may mimic the concentration of PF4 in PRP (Srivastava et al., 2010; Leiter et al., 2019). Via hair follicle organ culture, we treated the isolated human hair follicles with or without PF4 for 8 days. Our results showed that PF4 significantly suppressed hair shaft elongation in cultured HFs (Figs. 1A and 1B). Moreover, we found that PF4 inhibited the proliferation of epidermal cells in outer root sheath (ORS) of hair follicles (Figs. 1C and 1D).

### PF4 suppresses human dermal papilla cell proliferation

**Figure 2:**
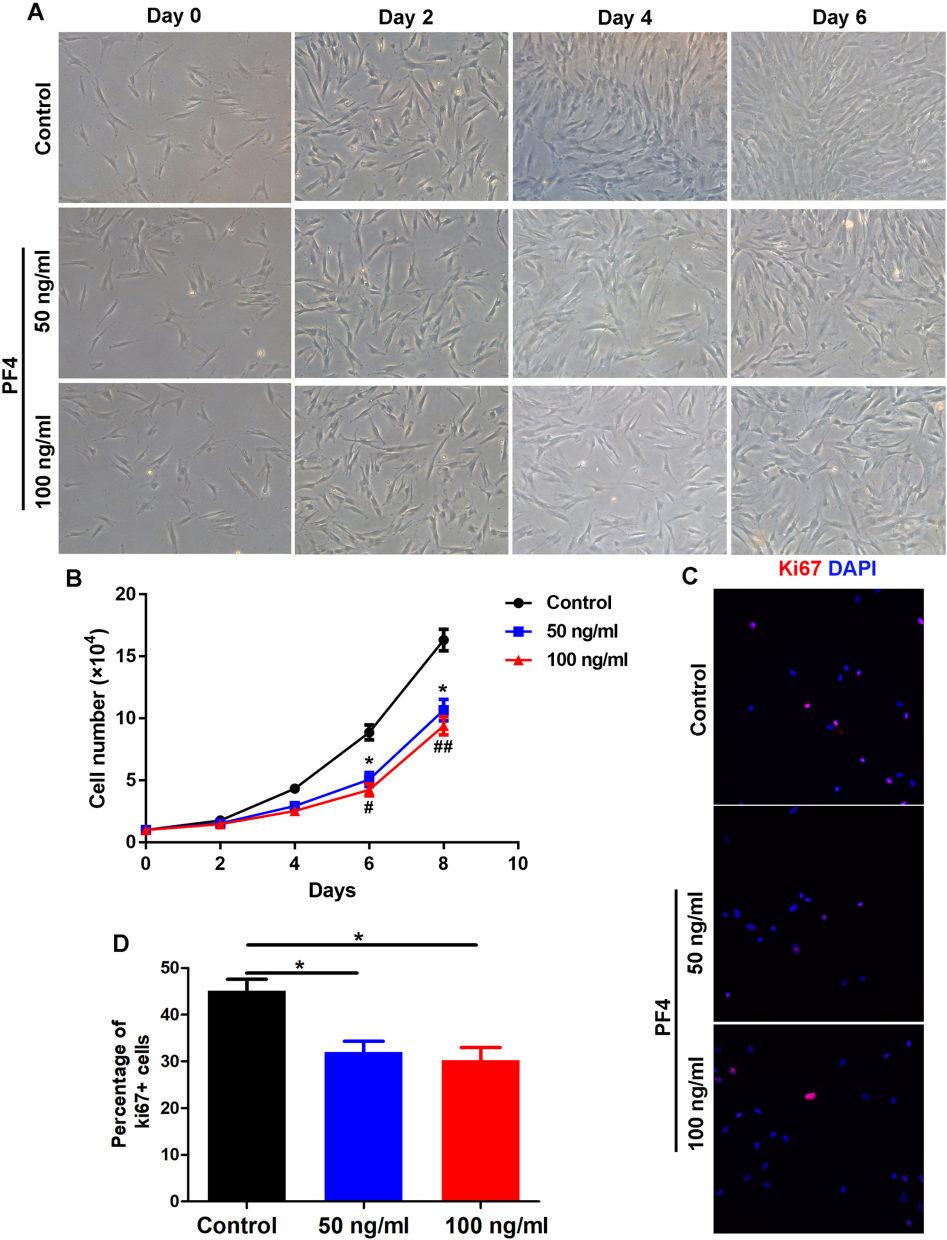
PF4 inhibits the proliferation of human DPCs. (A) Morphology of human DPCs treated with PF4 (0, 50, 100 ng/ml) at indicated days. (B) Human DPCs (1 ×10^4^cells) were plated in 12-well dishes and cultured in the presence of different concentrations of PF4 (0–100 ng/ml) for 8 days. Growth curves indicate the mean of three independent experiments (±SEM). (C) immunostaining of Ki67 in DPCs treated with PF4 (0, 50, 100 ng/ml) for 2 days. (D) Percentage of Ki67 positive DPCs. Student’s t-test was used to compare data. * *P* < 0.05,** *P* < 0.01, comparation between 0 and 50 ng/ml. # *P* < 0.05, ## *P* < 0.01, comparation between 0 and 100 ng/ml.

To determine the role of PF4 on the proliferation of cultured hDPCs, we administrated human dermal papilla cells with different doses of PF4 as described above. We showed that the quantity of hDPCs in the PF4-treated groups was obviously smaller than that in the control group, but the morphology of cells was not observably altered by PF4 treatment (Figs. 2A and 2B). By immunostaining of Ki67, a proliferative marker, we showed that PF4 repressed of DP cell proliferation *in vitro* (Figs. 2C and 2D). Collectively, these data suggest that PF4 inhibits the proliferation of hDPCs .

### PF4 increases the expression of androgen receptor in hDPCs

**Figure 3:**
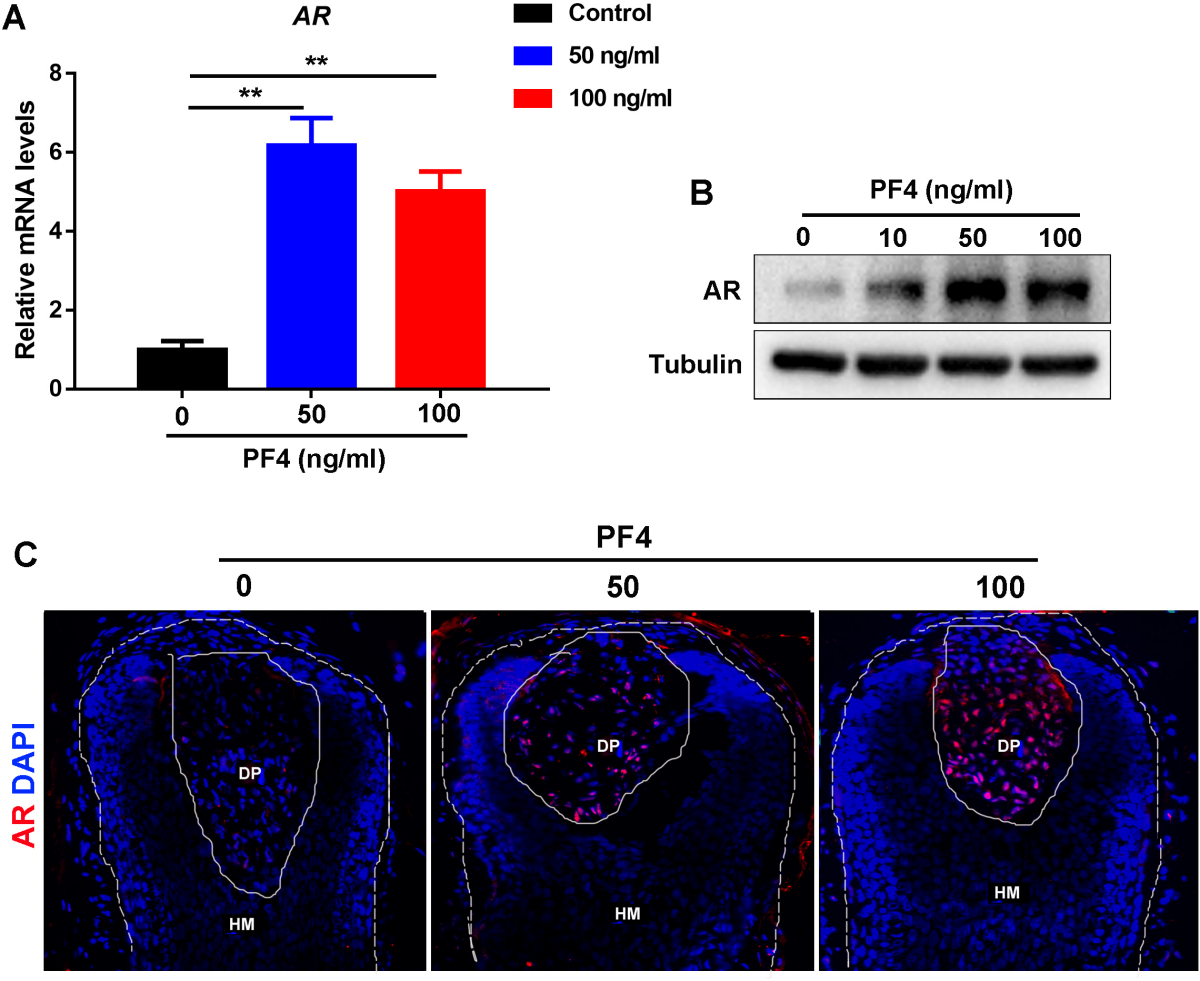
PF4 increases AR expression in human DPCs. (A) Effects of PF4 on AR mRNA expression in human DPCs cultured for 24 h. Data are reported as mean + SEM. Student’s t-test was used to compare data. ** *P* < 0.01. (B) Immunoblotting analysis of AR expression in hDPCs treated with PF4 for 48 h. Each experiment was repeated at least three times, and the typical blot was presented. Tubulin was used as loading control. (C) Immunostaining of AR in cultured human hair follicles treated with PF4 for 8 days. DP indicates dermal papilla; HM denotes hair matrix. DAPI staining (blue) indicates nuclear localization. Scale bar, 50 µm.

Previous studies have indicated that androgen receptor (AR) signaling in the DP cells plays a vital role in the modulation of hair follicle growth and development of androgenetic alopecia (Ceruti, Leiros & Balana, 2018), we wondered whether PF4 affects the expression of AR in the dermal papilla cells. Surprisingly, we found that PF4 treatment remarkably enhanced AR expression in hDPCs at both mRNA and protein levels *in vitro* (Figs. 3A and 3B). In addition, by immunostaining, we showed that PF4 also increased the expression of AR in DP cells in organ-cultured hair follicle (Fig. 3C). Overall, these results demonstrate that PF4 promotes the expression of AR in the DP cells.

### PF4 declines the hair growth-promoting properties of hDPCs

**Figure 4:**
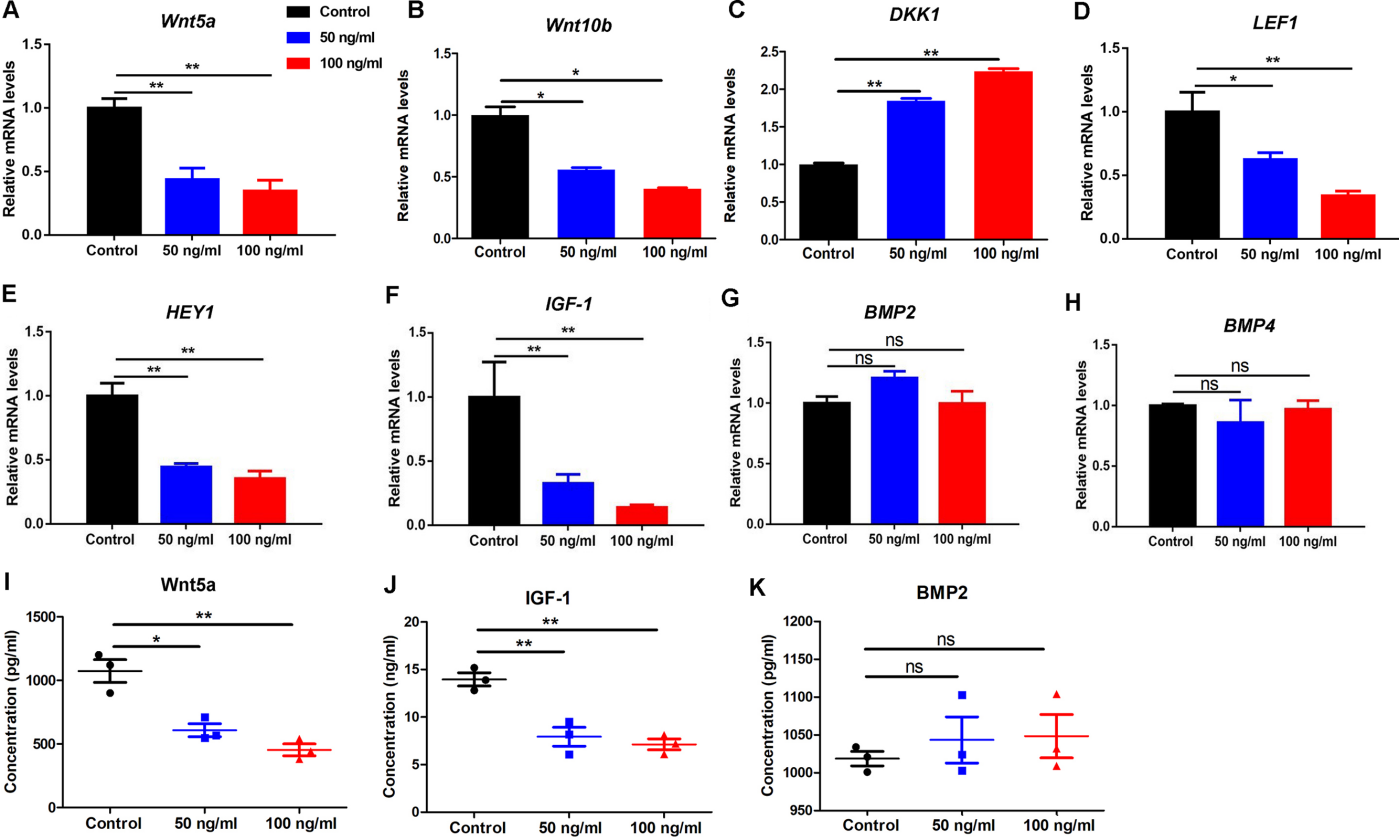
PF4 decreases the hair growth-promoting properties of hDPCs. (A–H) Effects of PF4 on hair growth-promoting and DP signature genes (*Wnt5a, Wnt10b*, *DKK1*, *LEF1, HEY1, IGF-1, BMP2 and BMP4*) mRNA expression in human DPCs cultured for 24 h. (I–K) Effects of PF4 on protein expression of *Wnt5a, IGF-1 and BMP2* in human DPCs cultured for 48 h. Data are reported as mean + SEM. Student’s t-test was used to compare data. * *P* < 0.05, ** *P* < 0.01. “ns” indicates no significant difference.

To further examine the effects of PF4 on human DP cells, we detected the expression of various hair growth-promoting and DP-signature genes. We showed that PF4 treatment significantly declined the expression of *wnt5a, wnt10b, LEF1, HEY1 and IGF-1*, and increased the expression o*f DKK1,* but did not affect *BMP2* and *BMP4* expression in hDPCs at mRNA levels (Figs. 4A–4H). By elisa assays, we further confirmed that PF4 upregulated Wnt5a and IGF-1, but did not altered BMP2 at protein levels in human DP cells (Figs. 4I–4K).

## Discussion

Platelet-rich plasma (PRP) derived growth factors (GFs) positively influence tissue regeneration. The GFs of PRP stimulated proliferation of adipose tissue-derived stem cells to accelerate wound healing (Scioli et al., 2017; Emer, 2019). On the basis that PRP can promote wound repair, PRP was employed to treat hair loss (Stevens & Khetarpal, 2019; Strazzulla et al., 2018). Recently, PRP has been demonstrated as an effective alternative treatment with minimal side-effects for hair disorders (Strazzulla et al., 2018), but the exact mechanisms and effects of specific components of PRP have not been uncovered. In this study, we showed that platelet factor 4 (PF4), a component of PRP, significantly inhibited human hair follicle growth and restrained the proliferation of human dermal papilla cells. Mechanically, our data demonstrated that PF4 promoted the expression of androgen receptor (AR) in human dermal papilla cells *in vitro* and via hair follicle organ culture, and decreased the expression of wnt5a, wnt10b, LEF1, HEY1 and IGF-1.

Though plenty of medicines are claimed to be effective for curing alopecia, the sexual-associated adverse-effects and efficiency are unpredictable (Varothai & Bergfeld, 2014; Rousso & Kim, 2014; Rogers & Avram, 2008). Recent evidence indicates that when injected into scalp, platelets in PRP become activated and release a number of growth factors and cytokines, such as PDGF), IGF-1 and EGF from their alpha granules, which may promote hair growth (Stevens & Khetarpal, 2019; Strazzulla et al., 2018; Cervantes et al., 2018). IGF-1 can stimulate proliferation of cycling Ki67 positive basal keratinocytes, and there is a higher number of Ki67 positive HFSCs in PRP-treated scalp tissue (Gentile et al., 2017; Cervelli et al., 2014). However, the precise effects of specific components in PRP on hair growth is still unclear. Surprisingly, in this study, we showed that PF4, a component of PRP, significantly inhibited human hair follicle growth via organ culture. To our knowledge, our study is the first to identify a component in PRP which suppresses human hair growth. This finding suggests that though PRP is an effective therapy for hair loss, it is necessary to further optimize this recipe, and neutralization of certain negative factors, such as PF4, may worth a shot. Previous studies have revealed that AR activity in the dermal papilla cells governs the hair cycle and plays an important role in the development of AGA (Ceruti, Leiros & Balana, 2018; Inui & Itami, 2011). We now demonstrate that PF4 increases the expression of AR in human DPCs both *in vitro* and in cultured human HFs, which might be one of the mechanisms by which PF4 inhibits human hair follicle growth. Previous studies have clarified that anagen phase duration determines the hair length, which has become shorter and eventually results in the progressive miniaturization and disappearance of hair in AGA (Varothai & Bergfeld, 2014), and anagen onset largely depends on wnt/ *β*-catenin signaling (Lien et al., 2014; Plikus & Chuong, 2014), whose activity in DP cells promotes the activation of HFSCs, thus triggering the growth of hair follicles (Morgan, 2014; Enshell-Seijffers et al., 2010; Lei et al., 2017; Lei & Chuong, 2016). Though PRP promotes hair follicle growth through wnt/ *β*-catenin signaling (Gentile & Garcovich, 2019), here our data show that PF4 significantly downregulated wnt-related genes in hDPCs. Previous study showed that androgen receptor could antagonize Wnt/ *β*-catenin signaling in skin cells (Kretzschmar et al., 2015). In this study, we found that PF4 increased androgen receptor expression and suppressed Wnt-related genes. We speculate that PF4 regulates Wnt/*β*-catenin signaling possibly via androgen receptor, but the precise mechanisms need to be figured out in the future.

Collectively, we report for the first time that PF4, a component of PRP which is an alternative therapy for AGA, significantly inhibits human hair growth via organ culture possibly through upregulating AR expression and decreasing hair-promoting genes. These data provide novel insights into the application and optimization of PRP in the treatment of hair disorders.

## Conclusions

Our findings suggest that PF4 inhibits human hair follicle growth probably by promoting androgen receptor signaling and downregulating hair growth-promoting genes, providing thoughtful insights into the application and optimization of PRP in hair loss treatment.

##  Supplemental Information

10.7717/peerj.9867/supp-1 Supplemental Information 1 qPCR primers

## Additional Information and Declarations

### Data Availability

The following information was supplied regarding data availability: The raw data is available at Figshare: Deng, Zhili (2020): Sha-2020-Rawdata.rar. figshare. Figure. https://doi.org/10.6084/m9.figshare.11828610.v1.

## References

[ref-1] Ceruti JM, Leiros GJ, Balana ME (2018). Androgens and androgen receptor action in skin and hair follicles. Molecular and Cellular Endocrinology.

[ref-2] Cervantes J, Perper M, Wong LL, Eber AE, Villasante FA, Wikramanayake TC, Jimenez JJ (2018). Effectiveness of platelet-rich plasma for androgenetic alopecia: a review of the literature. Skin Appendage Disorders.

[ref-3] Cervelli V, Garcovich S, Bielli A, Cervelli G, Curcio BC, Scioli MG, Orlandi A, Gentile P (2014). The effect of autologous activated platelet rich plasma (AA-PRP) injection on pattern hair loss: clinical and histomorphometric evaluation. Biomed Research International.

[ref-4] Chen M, Xie H, Chen Z, Xu S, Wang B, Peng Q, Sha K, Xiao W, Liu T, Zhang Y, Li J, Deng Z (2019). Thalidomide ameliorates rosacea-like skin inflammation and suppresses NF-kappaB activation in keratinocytes. Biomedicine & Pharmacotherapy.

[ref-5] Chen X, Chen R, Jin R, Huang Z (2020). The role of CXCL chemokine family in the development and progression of gastric cancer. International Journal of Clinical and Experimental Pathology.

[ref-6] Cieslik-Bielecka A, Choukroun J, Odin G, Dohan ED (2012). L-PRP/L-PRF in esthetic plastic surgery, regenerative medicine of the skin and chronic wounds. Current Pharmaceutical Biotechnology.

[ref-7] Deng Z, Chen M, Xie H, Jian D, Xu S, Peng Q, Sha K, Liu Y, Zhang Y, Shi W, Li J (2019). Claudin reduction may relate to an impaired skin barrier in rosacea. Journal of Dermatology.

[ref-8] Deng Z, Lei X, Zhang X, Zhang H, Liu S, Chen Q, Hu H, Wang X, Ning L, Cao Y, Zhao T, Zhou J, Chen T, Duan E (2015). mTOR signaling promotes stem cell activation via counterbalancing BMP-mediated suppression during hair regeneration. Journal of Molecular Cell Biology.

[ref-9] Emer J (2019). Platelet-Rich Plasma (PRP): current applications in dermatology. Skin Therapy Letter.

[ref-10] Enshell-Seijffers D, Lindon C, Kashiwagi M, Morgan BA (2010). Beta-catenin activity in the dermal papilla regulates morphogenesis and regeneration of hair. Developmental Cell.

[ref-11] Gentile P, Cole JP, Cole MA, Garcovich S, Bielli A, Scioli MG, Orlandi A, Insalaco C, Cervelli V (2017). Evaluation of not-activated and activated PRP in hair loss treatment: role of growth factor and cytokine concentrations obtained by different collection systems. International Journal of Molecular Sciences.

[ref-12] Gentile P, Garcovich S (2019). Advances in regenerative stem cell therapy in androgenic alopecia and hair loss: Wnt pathway, growth-factor, and mesenchymal stem cell signaling impact analysis on cell growth and hair follicle development. Cells.

[ref-13] Gledhill K, Gardner A, Jahoda CA (2013). Isolation and establishment of hair follicle dermal papilla cell cultures. Methods in Molecular Biology.

[ref-14] Gleissner CA, Von Hundelshausen P, Ley K (2008). Platelet chemokines in vascular disease. Arteriosclerosis, Thrombosis, and Vascular Biology.

[ref-15] Hattori H, Ishihara M (2017). Feasibility of improving platelet-rich plasma therapy by using chitosan with high platelet activation ability. Experimental and Therapeutic Medicine.

[ref-16] Inui S, Itami S (2011). Molecular basis of androgenetic alopecia: from androgen to paracrine mediators through dermal papilla. Journal of Dermatological Science.

[ref-17] Kretzschmar K, Cottle DL, Schweiger PJ, Watt FM (2015). The androgen receptor antagonizes Wnt/beta-Catenin signaling in epidermal stem cells. Journal of Investigative Dermatology.

[ref-18] Kwack MH, Sung YK, Chung EJ, Im SU, Ahn JS, Kim MK, Kim JC (2008). Dihydrotestosterone-inducible dickkopf 1 from balding dermal papilla cells causes apoptosis in follicular keratinocytes. Journal of Investigative Dermatology.

[ref-19] Lei M, Chuong CM (2016). Stem cells, Aging, alopecia, and stem cells. Science.

[ref-20] Lei M, Schumacher LJ, Lai YC, Juan WT, Yeh CY, Wu P, Jiang TX, Baker RE, Widelitz RB, Yang L, Chuong CM (2017). Self-organization process in newborn skin organoid formation inspires strategy to restore hair regeneration of adult cells. Proceedings of the National Academy of Sciences of the United States of America.

[ref-21] Leiter O, Seidemann S, Overall RW, Ramasz B, Rund N, Schallenberg S, Grinenko T, Wielockx B, Kempermann G, Walker TL (2019). Exercise-induced activated platelets increase adult hippocampal precursor proliferation and promote neuronal differentiation. Stem Cell Reports.

[ref-22] Li J, Jiang TX, Hughes MW, Wu P, Yu J, Widelitz RB, Fan G, Chuong CM (2012). Progressive alopecia reveals decreasing stem cell activation probability during aging of mice with epidermal deletion of DNA methyltransferase 1. Journal of Investigative Dermatology.

[ref-23] Lien WH, Polak L, Lin M, Lay K, Zheng D, Fuchs E (2014). In vivo transcriptional governance of hair follicle stem cells by canonical Wnt regulators. Nature Cell Biology.

[ref-24] Luo J, Chen M, Liu Y, Xie H, Yuan J, Zhou Y, Ding J, Deng Z, Li J (2018). Nature-derived lignan compound VB-1 exerts hair growth-promoting effects by augmenting Wnt/beta-catenin signaling in human dermal papilla cells. PeerJ.

[ref-25] Makarewicz-Wujec M, Henzel J, Kruk M, Kepka C, Wardziak L, Trochimiuk P, Parzonko A, Demkow M, Kozlowska-Wojciechowska M (2020). DASH diet decreases CXCL4 plasma concentration in patients diagnosed with coronary atherosclerotic lesions. Nutrition, Metabolism, and Cardiovascular Diseases.

[ref-26] Morgan BA (2014). The dermal papilla: an instructive niche for epithelial stem and progenitor cells in development and regeneration of the hair follicle. Cold Spring Harbor Perspectives in Medicine.

[ref-27] Mussano F, Genova T, Munaron L, Petrillo S, Erovigni F, Carossa S (2016). Cytokine, chemokine, and growth factor profile of platelet-rich plasma. Platelets.

[ref-28] Nicoli F, Balzani A, Lazzeri D, Gentile P, Chilgar RM, Di Pasquali C, Nicoli M, Bocchini I, Agovino A, Cervelli V (2015). Severe hidradenitis suppurativa treatment using platelet-rich plasma gel and Hyalomatrix. International Wound Journal.

[ref-29] Plikus MV (2012). New activators and inhibitors in the hair cycle clock: targeting stem cells’ state of competence. Journal of Investigative Dermatology.

[ref-30] Plikus MV, Chuong CM (2014). Macroenvironmental regulation of hair cycling and collective regenerative behavior. Cold Spring Harbor Perspectives in Medicine.

[ref-31] Premanand A, Reena RB (2018). Androgen modulation of Wnt/beta-catenin signaling in androgenetic alopecia. Archives of Dermatological Research.

[ref-32] Qi J, Garza LA (2014). An overview of alopecias. Cold Spring Harbor Perspectives in Medicine.

[ref-33] Rogers NE, Avram MR (2008). Medical treatments for male and female pattern hair loss. Journal of the American Academy of Dermatology.

[ref-34] Rousso DE, Kim SW (2014). A review of medical and surgical treatment options for androgenetic alopecia. JAMA Facial Plastic Surgery.

[ref-35] Schneider MR, Schmidt-Ullrich R, Paus R (2009). The hair follicle as a dynamic miniorgan. Current Biology.

[ref-36] Scioli MG, Bielli A, Gentile P, Cervelli V, Orlandi A (2017). Combined treatment with platelet-rich plasma and insulin favours chondrogenic and osteogenic differentiation of human adipose-derived stem cells in three-dimensional collagen scaffolds. Journal of Tissue Engineering and Regenerative Medicine.

[ref-37] Shin H, Yoo HG, Inui S, Itami S, Kim IG, Cho AR, Lee DH, Park WS, Kwon O, Cho KH, Won CH (2013). Induction of transforming growth factor-beta 1 by androgen is mediated by reactive oxygen species in hair follicle dermal papilla cells. BMB Reports.

[ref-38] Srivastava K, Field DJ, Aggrey A, Yamakuchi M, Morrell CN (2010). Platelet factor 4 regulation of monocyte KLF4 in experimental cerebral malaria. PLOS ONE.

[ref-39] Stevens J, Khetarpal S (2019). Platelet-rich plasma for androgenetic alopecia: a review of the literature and proposed treatment protocol. International Journal of Womens Dermatology.

[ref-40] Strazzulla LC, Avila L, Lo SK, Shapiro J (2018). An overview of the biology of platelet-rich plasma and microneedling as potential treatments for alopecia areata. Journal of Investigative Dermatology Symposium Proceedings.

[ref-41] Tang Y, Luo B, Deng Z, Wang B, Liu F, Li J, Shi W, Xie H, Hu X, Li J (2016). Mitochondrial aerobic respiration is activated during hair follicle stem cell differentiation, and its dysfunction retards hair regeneration. PeerJ.

[ref-42] Varothai S, Bergfeld WF (2014). Androgenetic alopecia: an evidence-based treatment update. American Journal of Clinical Dermatology.

