# Supplementary material for: Platelet factor 4 inhibits human hair follicle growth and promotes androgen receptor expression in human dermal papilla cells
Source: PeerJ. 2020 Sep 4;8:e9867. doi: 10.7717/peerj.9867 (PMC7476492; doi:10.7717/peerj.9867)
Supplement: Supplemental Information 1 [file peerj-08-9867-s001.docx]

| qPCR primers | |
| --- | --- |
| Human-*GAPDH*-F | TGTTGCCATCAATGACCCCTT |
| Human-*GAPDH*-R | CTCCACGACGTACTCAGCG |
| Human-*Wnt5a*-F | ATTCTTGGTGGTCGCTAGGTA |
| Human- *Wnt5a* -R | CGCCTTCTCCGATGTACTGC |
| Human- *LEF1*-F | GAATTAGCACGGAAAGAAAGA |
| Human- *LEF1*-R | ACCTGTACCTGATGCAGATT |
| Human- *HEY1*-F | GAAGTTGCGCGTTATCTGAGC |
| Human- *HEY1*-R | ATGCGAAACCAGTCGAACTCG |
| Human- *IGF-1*-F | GCTCTTCAGTTCGTGTGTGGA |
| Human- *IGF-1*-R | GCCTCCTTAGATCACAGCTCC |
| Human- *BMP2*-F | AGCAACGCTAGAAGACAGC |
| Human- *BMP2*-R | TGCTTCTTAGACGGACTGCG |
| Human- *BMP4*-F | TAGCAAGAGTGCCGTCATTCC |
| Human- *BMP4*-R | GCGCTCAGGATACTCAAGACC |
| Human-*AR*-F | CCAGGGACCATGTTTTGCC |
| Human-*AR*-R | CGAAGACGACAAGATGGACAA |
| Human-*DKK1*-F | CCTTGAACTCGGTTCTCAATTCC |
| Human-*DKK1*-R | CAATGGTCTGGTACTTATTCCCG |
| Human-*Wnt10b*-F | CATCCAGGCACGAATGCGA |
| Human- *Wnt10b* -R | CGGTTGTGGGTATCAATGAAGA |
|  |  |
